# Difficulties with clinical practice guidelines for congenital syphilis prevention in Colombia: a qualitative study

**DOI:** 10.1186/s12879-026-12652-9

**Published:** 2026-01-29

**Authors:** Ana Estrada-Jaramillo, Mike Michael, Hannah Farrimond

**Affiliations:** 1 Anthropology and Ecology of Disease Emergence, Institut Pasteur–Université Paris Cité, 25 Rue du Dr Roux, Paris, 75015 France; 2 Social and Political Sciences, Philosophy and Anthropology, University of Exeter, Rennes Drive, Exeter, EX4 4RJ UK; 3 Egenis, University of Exeter, Saint German’s Road, Exeter, EX4 4PJ UK

**Keywords:** Congenital syphilis, Prevention, Colombia, Clinical practice guidelines, Migrants, Indigenous, Afro-descendants

## Abstract

**Background:**

Congenital syphilis (CS) is considered a preventable disease. However, it remains a major public-health concern in Colombia, where healthcare authorities have suggested that the main issue is the incorrect implementation of the clinical practice guidelines (CPG). This study aimed to understand why this preventable disease cannot be prevented in Chocó and Caldas, two regions of Colombia.

**Methods:**

A qualitative online study, using a multi-method approach following interpretative and ethnographic online research principles, was conducted in two regions of Colombia (Chocó and Caldas) during the COVID-19 pandemic.

**Results:**

Besides the difficulties of, and barriers to, implementing the CPG identified in other studies in Colombia and elsewhere, three main gaps are highlighted as results of the study. (1) The CPG is usually implemented in a fragmented system. It specifies timelines and trajectories to be met in dynamic, inter-connected and under-resourced systems. (2) The CPG’s implementation requires measures and activities that go before and beyond prenatal care and involve more than pregnant women and healthcare workers. (3) The CPG are for preventing the specific disease, but the implementation is enacted in relation to other diseases (HIV, COVID-19, hepatitis B, hypertension, diabetes) and their protocols and clinical guidelines.

**Conclusions:**

The implementation of the CPG is important but insufficient for CS prevention. The CPG does not operate in isolation. In addition to the knowledge of the CPG and the provision of the necessary resources, it is crucial to consider the activities and processes before and after prenatal care to address the fragmentation of the healthcare system and healthcare services provision. Moreover, it is essential to coordinate the activities and practices for other diseases (HIV, COVID-19, hypertension, hepatitis B, diabetes) with those of the CPG.

## Background

Congenital syphilis (CS), the transmission of mother-to-child syphilis is considered a preventable disease, insofar as syphilis in pregnant women and their sexual contacts can be readily diagnosed and treated. Since 2014, clinical practice guidelines (CPG) indicating the screening of pregnant women during prenatal care, the use of rapid tests and same-day treatment at the point of care have been introduced in Colombia [1]. Despite the implementation of the guidelines, CS has not decreased in Colombia [2].

Several difficulties for CS prevention are indicated in the literature, for instance, the provision of penicillin in a primary care setting [3], the rapid test sensitivity and availability [4], the execution of timely and effective prenatal care appointments [5], and sexual contact’s treatment provision [6].

On the other hand, Colombian health authorities have identified the incorrect implementation of the CPG as the major hindrance to CS prevention [7].

Research on CPGs in relation to a diverse range of diseases and in various countries has highlighted challenges for their implementation [8, 9]. Studies in Colombia have indicated that healthcare workers’ lack knowledge of the CPG [10, 11] and administrative barriers and difficulties with healthcare insurance companies [12, 13] are the major obstacles to CPG implementation and CS prevention in Colombia.

There are two types of healthcare insurance in Colombia. A contributory insurance for those with payment capacity, and a subsidised insurance funded by the government’s fiscal resources and cross-subsidies from contributory insurance. Healthcare service administrators (EPS is the acronym in Spanish) receive contributions from both insurance systems and establish contracts with healthcare service providers (IPS): hospitals, clinics, and laboratories. Healthcare service provision can be affected by administrative reasons, such as changes in affiliation type from contributory to subsidised due to job loss or lack of EPS coverage in certain regions.

Several studies have emphasised the fragmentation of care in Colombia [9, 14]. This fragmentation not only refers to the discontinuity in health service provision among different medical specialists, but also the lack of integration among healthcare providers. The studies show delayed service provision, adverse health outcomes and differences among regions, highlighting the need for coordination and integration of healthcare [15].

The present research explores why a preventable disease such as CS has not been prevented, or at least not prevented as expected.

Rather than focusing on the lack of knowledge of the CPG or the individual values and attitudes of healthcare workers and pregnant women towards the CPG and CS prevention, the study focuses on practices.

Recent developments in practice theory [16] have emphasised the necessity to analyse action in context, where instead of focusing on lifestyles and individual behaviours or decision making for behavioural change, the focus is on activities where routines, objects, infrastructures, institutions and collective norms and interdependence with other activities configure practices [17].

A “practice” is an “organised form of activities” [18] that involves materials (objects, infrastructures), competences (understandings of the situation, know-how) and meanings (significance of the practice and past experiences). An analysis of practices requires, on one hand, attention to the “nexus of connections” [19] with other elements and practices, and on the other hand, how practices remain together in the present and matter for future interactions [16, 20].

A considerable number of studies have integrated this approach [21–23], particularly regarding electricity and food consumption, to identify potential interventions. Others [17, 24] have explored how practice theory can contribute to understanding drinking, smoking and addiction in order to change those practices.

## Methods

The qualitative multimethod [25] online study [26] was conducted during the COVID-19 pandemic, between January and August 2021. The Consolidated Criteria for Reporting Qualitative Research (COREQ) guidelines [27] have been used for reporting.

The present research encompassed documentary evidence and qualitative data collection with healthcare workers and administrators, pregnant women or recent mothers and their partners from Quibdó, Lloró (Chocó) and Manizales, Riosucio (Caldas). These two regions have different ethnic, social and political characteristics, Chocó being one of the poorest regions with the highest incidence of CS in Colombia [28, 29]. Most of Chocó’s population self-identifies as Afro-descendant, but there are also indigenous groups – namely, the Emberá, Tule, Cuna, and Wuanan people. In contrast, in Caldas, the majority self-identifies as “without ethnic affiliation”, according to the last census [30].

The research participants were between 19 and 70 years old, with the age range of 19–34 years, specifically among pregnant women and recent mothers. The participants self–identified as mixed background, Indigenous (Embera Chami), and Afro-descendants. There were two international migrants and one Internally Displaced Person from the armed conflict (see Table 1).

**Table 1:** Characteristics of participants

Participants	Number
Healthcare workers	6 (1 male)
Healthcare administrators	10 (2 males)
Researchers	2 (females) national level
Pregnant women and recent mothers	Interviews (27) OAFG (19), (3) diaries (all females)
Partners	2 (males)
Traditional midwives	3 (females)
Documents	7

We recruited participants through snowballing, including referrals from the Secretaries of Health, an Association of Traditional Midwives in Chocó (ASOREDIPARCHOCO), an NGO in Manizales (Sagrada Familia), and other participants. For the document analysis, criterion sampling [31] was employed for the documents most relevant to CS prevention.

### Design and setting of the study

The study included document analysis of the CPG [1, 32], (particularly CS, pregnancy and birth complications); decrees and laws; 47 semi-structured interviews; 3 online asynchronous focus groups (OAFG), with 19 participants in total; and 3 diaries written by pregnant women.

The rationale behind the multimethod approach rests on the benefits that each method provides for the study. The documents offer insights into organisational routines [33], knowledge and implementation practices [34]. In addition, semi-structured interviews offer insights into the participants’ experiences [35]. The potential of focus groups lies in their ability to facilitate the discussion of sensitive topics [36] and the inclusion of underrepresented groups [37, 38]. Besides allowing access to personal experiences, diaries are also beneficial when participants have privacy concerns [39–41], as was the case during the COVID-19 pandemic.

### Description of processes

All potential participants received an information sheet about the research project, complemented by an infographic for the pregnant women and recent mothers and an informed consent form. Eight healthcare workers and administrators refused to participate and one recent mother dropped out from the OAFG during the second day. In both situations, tight work schedules and responsibilities were the reasons for their non-participation.

The OAFG and diaries were conducted through WhatsApp, and the interviews were conducted through WhatsApp or mobile phones.

The document analysis, interviews, diaries and OAFG were conducted in Spanish by (AEJ) (a female anthropologist with extensive experience in qualitative research), who already knew some of the participants from her previous work on barriers to, and facilitators of, the implementation of CPG in Cali (Colombia).

Due to the COVID-19 pandemic restrictions, the interviews, OAFG, and diaries were conducted online at participants’ home, workplaces or public space. It is not possible to identify with certainty whether other people (besides the participants) were present during those times, but in five interviews and two OAFGs, background noises could be heard and some participants made comments in this regard.

The semi-structured interviews were audio-recorded on a digital recorder by AEJ and lasted between 25 and 80 min. They included questions regarding prenatal care, pregnancy experiences, difficulties with implementing the CPG, and CS prevention.

Three WhatsApp groups were set up as OAFGs over three days, starting at 8:30 a.m. and finishing by 6:00 p.m. Each group had between 6 and 7 participants, and between 8 and 10 questions in total were prompted in the chat, one every two hours. Interactions between participants and the moderator (AEJ) included text messages, voice messages, emojis, stickers, links, and songs. Seven interviewees and two diary participants also participated in the OAFG. More information on the OAFGs can be found at [42].

Three pregnant women shared their thoughts, feelings and opinions about their experiences of pregnancy between March and August 2021 by journaling for at least one week in every trimester.

Following the recommendations of healthcare workers and administrators, seven key documents were selected and the connections between procedures, laws, decrees and administrative requirements for the implementation of the CPG were analysed. The documents were the CPG for gestational syphilis (GS) and CS [1], the CPG for pregnancy and birth complications [13], Law 1751 of 2015 in Health (Intercultural Health Law), *Protocolo de Atención Preconcepcional* [Preconception Counselling Protocol] [43], *Sistematización de experiencias de parteras ASOREDIPARCHOCÓ* [Systematisation of experiences], and reports from National Institute of Health regarding CS and GS prevalence during 2020 and 2021 [28, 29].

The interviews, OAFGs and diaries were verbatim transcribed in Spanish by AEJ (Spanish native speaker), following the conventions for conversation analysis [44]. Special emphasis was placed on including silences, nonverbal cues, background noises, text, emojis, stickers, and links shared in the OAFGs. The transcripts of the interviews, OAFGs and diaries were not returned to participants for comments or corrections.

Fieldnotes were also considered for the analysis, including contextual information and reflection on emails, calls, text exchanges and descriptions before, during, and after the interviews, OAFGs, document analysis and diaries. All the data was initially analysed in Spanish by AEJ. After AEJ derived the themes, these were shared with relevant translated data in English by AEJ (C2 English level) and double checked for sense and robustness with English native speakers MM and HF for further analysis.

### T*ype of analysis*

**Fig. 1:**
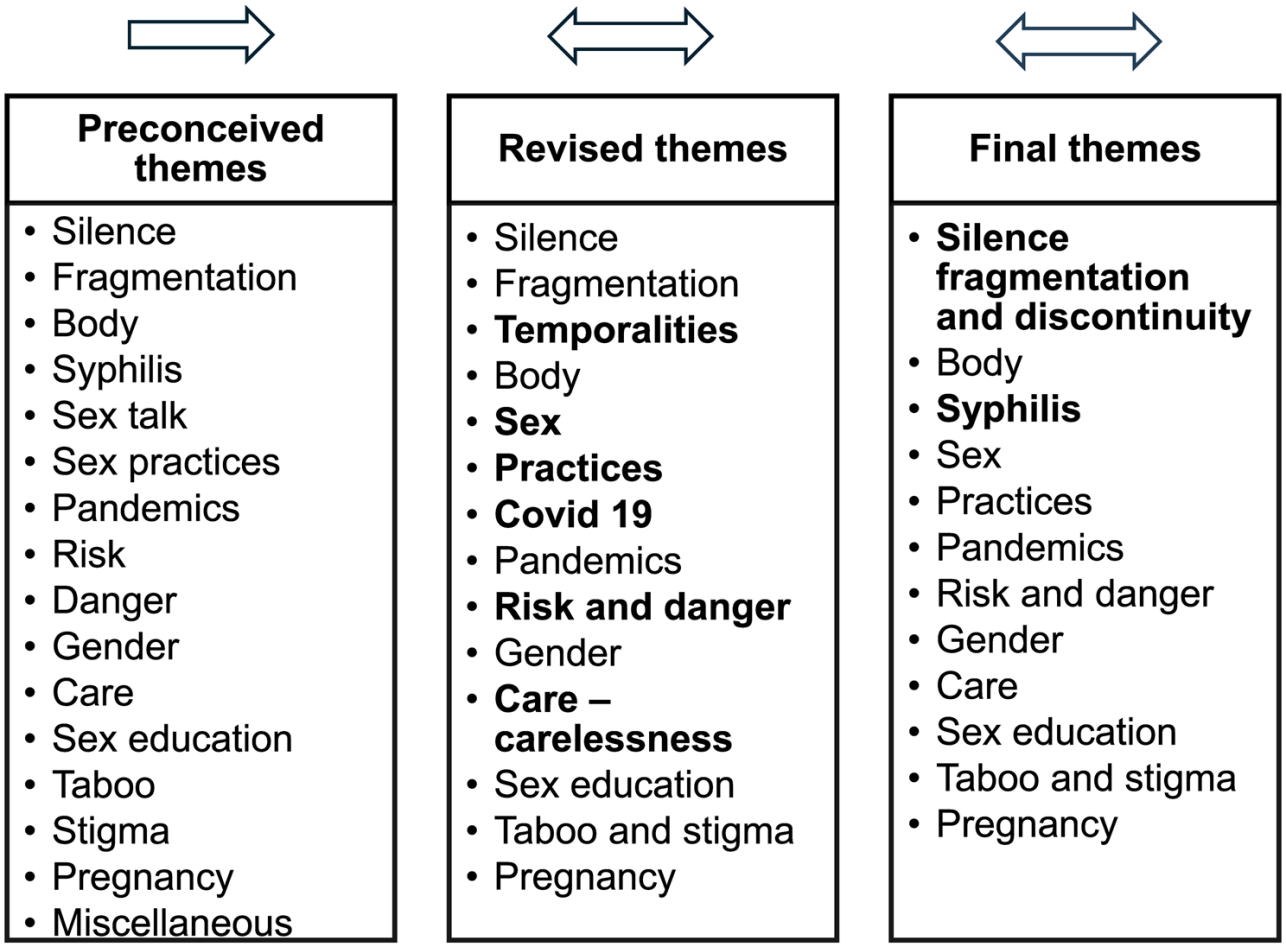
Thematic analysis

The research involved an iterative process [26], alternating between inductive and deductive strategies. After we (MM, HF, AEJ) had identified themes, processes and interactions during the initial document analysis and familiarised ourselves with the data, a set of themes and categories for each type of participant was identified from the interviews, diaries, documents and OAFGs, following the principles of thematic analysis [45, 46] (Fig. 1 preconceived themes). One of these (“miscellaneous”) included categories and sub-themes that seemed to lack a clear connection with others. Contrary to the accustomed tendency to seek consensus in thematic analysis (i.e. grounded theory), a search for contradictions, controversies and negotiations was followed, as proposed by McGaw and Vance [47]. Discussions held by the authors (MM, HF, AEJ) around categories continue as they deepen the analysis, leading to some emergent themes and categories as shown in Fig. 1 (revised themes). The discussions held monthly between August 2021 and June 2023 were conducted remotely, with notes and minutes prepared by AEJ and agreed with MM and HF. The focus of those discussions was initially situating the data and themes within the broader empirical field (Colombian health system, cultural framings, social settings etc.), and the broader conceptual field (science and technology studies, medical sociology and anthropology).

Using mind maps and discussions, the authors (AEJ, MM, HF) identified 12 final themes and categories (Fig. 1), following reiterative patterns and theoretical questions from the research. The themes were silence - fragmentation and discontinuity; the body; syphilis; sex; practices; pandemics; risk and danger; gender; care; sex education; taboo and stigma; and pregnancy. These refined themes were contrasted and analysed against the data.

Figure 1 Coding process. The figure displays the coding process according to Iterative Thematic Analysis [29]. Emergent categories are in bold, showing the analytical process.

In this research, the multimethod approach, beyond secure triangulation [31], enabled the exploration of practices and interactions without requiring closure, highlighting the multiplicity, fluidity, and emergence of realities [48, 49].

## Results

Through the analysis, several difficulties related to the implementation of the CPG and CS prevention reported by the literature were also found in the interviews, OAFG, diaries and documents. Appropriate and timely prenatal care, information on STIs during pregnancy, test availability and sensitivity, the provision of penicillin in a primary care setting due to potential adverse reactions, and the provision of treatment to sexual contacts were consistently highlighted.

Nevertheless, the results presented here focus mainly on three themes that particularly illuminate the difficulties arising during the implementation of the CPG that are limiting CS prevention.

The data analysis showed underlying assumptions in the design and implementation of the CPG. In particular, the guidelines aimed to integrate institutions, people, procedures, laws, and activities at specific trajectories and timelines. However, the data also revealed that the CPG is not implemented in isolation and, rather, requires the coordination of processes, practices, materials, institutions and people for its implementation. In what follows, to reiterate, three broad themes are highlighted that both provide insights into the shortcomings of the CPG, but also can facilitate specific actions for a potentially better implementation of the CPG and disease prevention. Those three themes concern the configuration of the healthcare system, the span of CS prevention, and the connection of CS with other diseases.

1. The CPG is implemented in a healthcare system that is usually fragmented.

The implementation of the CPG requires precise times and trajectories. Women are supposed to start prenatal care before the tenth week of pregnancy and are expected to attend at least seven appointments before giving birth. Besides information on their physical and psychological state collected in prenatal cards and medical records, several screenings, including for syphilis, are run during prenatal care appointments.

According to the CPG [1], women should be screened with a rapid syphilis test during the first prenatal care appointment, in the second trimester, and before giving birth. If a positive result is obtained, the first dose of penicillin is administered at the point of care if there is no history of allergic reaction. A treponemal test (VDRL or RPR) is also taken to establish reactivity and for follow-up. Three doses are provided (one weekly) to the pregnant woman, and at least one is given to her partner.

The continuity and linearity needed to complete these activities are – the participants made clear – constantly interrupted. A healthcare worker indicated that pregnant women start their prenatal care “late”:

*It is common that pregnant women start prenatal care during the third trimester*,* 30 weeks of pregnancy approximately*,* and they do not have a complete treatment… there are several factors for that [starting prenatal care late]*,* and one of the reasons is cultural. They think prenatal care must start after the third month of pregnancy. We tell them that they should start prenatal care as soon as they realise they are pregnant. But they prefer to stay at home*,* and during the pandemic*,* they don’t want to go to the hospital (Interview 3*,* Nurse prenatal care).*

Another reason to start late prenatal care is that the pregnancy was unplanned. Only two participants in the study had preconception counselling; for most of them, the pregnancy was unexpected. Access to contraception during the pandemic was difficult for four participants, as was also the case for other women in Colombia [50].

There are also difficulties in healthcare access related to the characteristics of the Colombian healthcare system.

In this study, the data also showed high mobility in the population in Colombia (between municipalities, regions, types of job contracts, temporary jobs, healthcare insurance, and EPSs). This suggests difficulties regarding access to healthcare services. Information regarding insurance affiliation, medical records, lab tests, and treatment is not integrated into different parts of the healthcare system. As explained by several research participants:

*… These institutions buy their own software*,* so each patient comes with a printed* Medical record from each hospital that she has attended. Usually, these patients have their prenatal care at the first level. They rarely end up at the third level for syphilis. Sometimes it is because of something else – like early birth, preeclampsia… Even in hospitals with the same software, we cannot see the medical records from *another hospital (Interview 37*,* Healthcare worker).*

*… the GP nagged me because I didn’t have the medical records*,* I didn’t have the test results*,* and because I didn’t know what they had done to me (Interview 23*,* Pregnant woman).*

The contract with an IPS depends on the EPS and its regional coverage, which might, in some cases, result in the partner of a pregnant woman with a positive diagnosis not receiving treatment if the two individuals belong to different EPSs.

There are also limitations on service provision according to migratory status (particularly for undocumented people), despite the efforts made by the government and other organisations to provide healthcare services to migrants.

*The year before last year (…) 30% of gestational syphilis cases occurred among* Women without healthcare insurance. 90% of those were undocumented migrants From Venezuela. So, that is difficult (…) there are people who don’t have insurance *because they don’t have a job*,* right? But they also don’t have money to pay. (Interview 7*,* Healthcare administrator).*

The integration between healthcare services and the transmission of information is an important function of prenatal cards. However, this is not necessarily accomplished during prenatal care. Healthcare workers interviewed, privileged information recorded on the medical record regarding syphilis diagnosis and treatment over the information on prenatal cards. Even if medical records are used, they pose difficulties regarding healthcare integration due to the different formats (paper, digital), the software used across medical settings, and, consequently, the software restriction policies imposed on users.

Additionally, the need for multiple authorisations for service provision and the fragmentation of care impose yet more barriers to healthcare access. All the pregnant women and recent mothers interviewed had been required to access health services at distant sites, some even outside their municipality. Some of these difficulties had been exacerbated by the COVID-19 pandemic, particularly during the first months of lockdown, when travel and economic restrictions also played a role. For the CPG to be adequately implemented, more fluid financial and administrative systems are required in order to limit the barriers and borders imposed by the classification and demarcation between types of health insurance, types of affiliation, regions and municipalities.

For example, it is paramount that there is correspondence between contributory and subsidised health-insurance systems, given the movement of people between types of health insurance and regions. Similarly, the systems’ integration will need to accommodate a constant flux of information on health-insurance affiliation, medical records and lab tests that should flow between institutions and health practitioners to facilitate prenatal care and the prevention of CS.

2. The CPG for the prevention of CS requires other measures and activities that take place before and beyond prenatal care, involving others in addition to pregnant women and healthcare workers.

Besides assuring resource availability (syphilis rapid tests, penicillin, the necessary equipment to assist in case of an adverse reaction at a point of care) and competencies (knowledge of syphilis, the CPG, the interpretation of test results), other measures and activities are also necessary. For example, it is important to provide sex education, beyond at-risk or sexually active people, to the general population. One pregnant woman – along with many other interviewees – explained that, typically, no sex education is provided at all:

*At home? No*,* we never talked about those things [about sex] or at school. We didn’thave a teacher to talk to about it*,* no … {how did you learn} Well*,* to be honest*,* having sex with your boyfriend [was how you learned about sex] but we didn’t talk about it… We talked with our friends and shared our experiences and things like that. (Interview 36*,* Pregnant woman).*

Similarly, a healthcare administrator highlighted:

*…we have to teach people how to use condoms and dental dams*,* explain what happens with lubricants*,* what happens with oral sex and anal sex*,* and what happens with sex toys. We have to talk about it because there are many options*,* and there are risks associated with those things. But we don’t [talk about it] and we should. (Interview 18*,* Healthcare administrator).*

Sex education is intended to limit the taboos around sex, sex talk (especially during pregnancy), STIs and their prevention.

In this regard, interviewed healthcare workers and administrators emphasised the relevance of activating syphilis screening for the general population, which is not only directed to at-risk groups and pregnant women. They also indicated that there were inconveniences implicated by the syndromic management of STDs as recommended by WHO, as well as the potential of this to lead to antimicrobial resistance.

This was highlighted by several healthcare workers and exemplified in the following testimony:

*…something that has to change is the syndromic treatment…in countries like ours*,* we have good labs*,* and STDs are common…. someone can have a genital ulcer*,* and it can be syphilis*,* but it is not diagnosed as syphilis; it is diagnosed as genital ulcer… or you can have a patient with urethral discharge*,* you will never know if it was chlamydia or gonorrhoea*,* if it is gonorrhoea resistant*,* nothing! You can’t know anything (Healthcare worker*,* Interview 12).*

A combination of sex education and STI screening directed to the general population would encourage people to relate to their own bodies – and those of others – in different ways and promote other disease-prevention practices. Knowledge of one’s own body allows people to identify signs and symptoms that usually go unnoticed. It also encourages people to ask questions and talk openly about those signs and symptoms, and can help with identifying pregnancy and STD diagnoses in the early stages. General population screening can also identify cases at early stages and potentially reduce stigmatisation if accompanied by sex education.

3. The CPG for CS prevention is not isolated, but interconnected with the prevention of other diseases, such as HIV, COVID-19, hypertension, diabetes and hepatitis B, each of which has its own CPG. It is crucial to consider these connections, particularly when providing care to pregnant women and their babies during the first months.

During the COVID-19 pandemic, there was more emphasis on COVID-19 (prevention, diagnosis and treatment), which had the effect of diminishing the time and resources set aside for CS prevention. Healthcare workers and administrators dedicated more effort to the pandemic; those who would usually carry out CS prevention activities also had to support the COVID-19 pandemic response.

Healthcare workers and administrators tend to focus more on risks associated with diabetes, hypertension and anaemia during pregnancy, rather than signs of syphilis. During prenatal care, in pamphlets, and even during the interviews, there were constant comments about “warning signs” and what to do in case of those signs, as exemplified in the following testimony:

*If I bleed*,* have lower pain or pain that doesn’t stop*,* or contractions for more than* four minutes, or unbearable headaches or earaches or blurry vision (…) or if I don’t *feel the baby” (Interview 24*,* Pregnant woman).*

Among pregnant women or recent mothers, syphilis was not well known in comparison to HIV. Several of the pregnant women who were interviewed asked what syphilis is, as they had heard (and knew) little about it. In comparison, three of the pregnant women who were interviewed recalled that some of their relatives had experienced HIV. In the words of one interviewee:

*What is syphilis? … I had an HIV test [during prenatal care]*,* and they told me that if it is positive*,* the psychologist will explain to me the risks*,* and they would also test the baby after birth (Interview 11*,* Recent mother).*

In line with other STIs, syphilis also carries stigma that becomes greater for pregnant women because they are usually considered responsible for the outcomes (i.e. CS in their babies). There is a close relationship between HIV and syphilis, as both can be asymptomatic and go unnoticed for a period of time. There is also a proportion of co-occurrence between HIV and syphilis, which further complicates their treatment. The use of rapid dual tests (HIV and syphilis) has been seen as an advantage, particularly during prenatal care screening. Nevertheless, more attention is directed to HIV prevention.

One healthcare interviewee said,

*…to me*,* syphilis is like the Cinderella of all the diseases. I don’t know why*,* but we can see that other diseases have a lot more resources. I have spoken about it*,* but there was no echo… Penicillin is cheap and there are so few exams for its diagnosis*,* but no. I don’t know why. (Interview 18*,* Healthcare administrator).*

Even if other conditions like maternal mortality can have higher prevalence compared to gestational syphilis and CS, the latter continues to be a public health concern in Latin America, and lately, in high-income countries where it was not traditionally a concern [51]. Historically, several goals for CS reduction and elimination have been established, highlighting CS as a preventable disease that does not pose difficulties in its treatment (cost, technology, or effectiveness). It is precisely by researching CS and the CPG that this study aims to contribute to their visibility and relevance.

CS prevention would benefit from measures also considered for preventing other diseases, including those related to mother-to-child transmission. These include measures to overcome financial and administrative barriers to healthcare service provision, as well as test screening, sex education, and knowledge of signs to look for beyond those considered ‘warning signs’.

## Discussion

Besides knowledge of the CPG and the limitations imposed by barriers to its implementation, as highlighted by studies conducted in Colombia [6, 8], it is crucial to address how the CPG is implemented in a context in which the healthcare system is fragmented [9] and where the timing by which women engage with the relevant services is highly variable. As indicated by other studies [52], even if resources are allocated and sufficient knowledge and training are provided, this does not mean that the recommendations of CPG can be enacted. Moreover, as shown by research conducted in Brazil [53], increased access to and testing during prenatal care do not necessarily translate into a reduction in CS. Reduction of syphilis incidence and prevalence during pregnancy as well as in the general population is also needed. The increase in syphilis worldwide and among certain groups (men who have sex with men, MSM) [54] also implicates the necessity of coordinated efforts for syphilis control besides prenatal care.

The CPG should not be considered a tool or device that functions in isolation. In fact, the CPG requires coordination between activities, processes, institutions, objects, infrastructures, people and communities. Therefore, it is crucial to involve more patients and communities during the design and implementation of the CPG to provide a better contextualisation of the implementation of the CPG, as it has also been highlighted by [55].

Identifying connections between several CPGs and disease prevention is necessary, as diseases do not occur in isolation, and various measures can be taken to ensure the implementation of both CPGs and disease prevention. This is in line with other studies [9] that have identified that the multiplicity of CPGs is a barrier to their implementation.

### Limitations of the study

The Covid-19 pandemic affected the recruitment of participants, particularly healthcare workers, who had to manage Covid-19-related activities (prevention, diagnosis, surveillance) alongside their usual duties. This was particularly the case in the region of Chocó, where GPs did not participate.

Contrary to classical ethnographic studies, the observation of practices was limited to online interactions. Some non-verbal and contextual information is missing, including some routinised activities, workarounds or implicit norms.

Nevertheless, by considering practices as a mix of actants, objects, discourses and technologies that perform a particular reality [48], other data (including sounds, silences, connections between processes and actions, and absences) provided rich insights. The mix of interviews, OAFG, diaries and documents provided complementary and contrasting elements regarding the experiences and conditions of prenatal care and the implementation of the CPG according to HCW, healthcare administrators and pregnant women. It leads to constantly reflecting on what was missing, what was covered, and what was made absent.

The translation of data from Spanish to English, made by a proficient English speaker and double-checked by native English speakers, nonetheless implies that some sense is lost in translation.

The COVID-19 pandemic brought additional difficulties for the implementation of the CPG, limiting the range of the conclusions from the present study to non-pandemic contexts. Nevertheless, several conditions, situations and practices were and are still present after the pandemic. Difficulties in accessing healthcare services [56] especially for migrants [57, 58], and fragmentation in care provision [13, 14] continue in Colombia.

The study does not represent the diversity of regions in Colombia and the multiple ethnic groups, diverse administrative procedures, and economic characteristics (with considerable variations in inequality). Moreover, since there is variability between the CS case definitions and the diagnosis algorithms used for treatment (traditional or reverse), variability that further manifests differences between high-income and low- and middle-income countries [59], the conclusions might only apply to some contexts.

Furthermore, this study does not account for the implementation of the EMTCT Plus strategy developed by the Pan American Health Organisation [60] to prevent several mother-to-child transmitted diseases, since it had yet to be implemented in these regions at the time the fieldwork was conducted.

## Conclusions

While implementing the CPG is important, it is insufficient for CS prevention. The CPG does not operate in isolation. In addition to promoting knowledge of the CPG and providing the necessary resources for its implementation, it is crucial to consider the activities and processes that occur before and after prenatal care, when the CPG is typically implemented. It is also essential to address the fragmentation of the healthcare system and the provision of healthcare services. To improve CS prevention, it is paramount to coordinate activities across diverse institutions and practices involving healthcare workers, pregnant women and their families. The engagement of patients, institutions, communities, and leaders during the design and implementation of the CPG might contribute to its contextualisation and coordination in specific settings.

## Data Availability

The data presented in this study is available on request, subject to authorisation by the University of Exeter ethics committee. Requests can be sent to the corresponding author at: ana-lucia.estrada-jaramillo@pasteur.fr.

## Abbreviations

CPG: Clinical practice guidelines
CS: Congenital syphilis
GS: Gestational syphilis HIV: Human immunodeficiency virus
HIV: Human immunodeficiency virus
STI: Sexually transmitted infection
STD: Sexually transmitted disease
OAFG: Online asynchronous focus groups
ASOREDIPARCHOCO: Association of traditional midwives in Chocó
NGO: Non-governmental organisation
EPS: Healthcare service administrators (acronym in Spanish)
IPS: Healthcare service providers (acronym in Spanish), VDRL: Venereal disease research laboratory test
VDRL: Venereal disease research laboratory test
RPR: Rapid plasma regain
PAHO: Pan American Health Organisation
EMTCT Plus: Framework for elimination of mother-to-child transmission of HIV, syphilis, hepatitis B and Chagas
WHO: World Health Organization
